# Overcoming Challenges of Lignin Nanoparticles: Expanding Opportunities for Scalable and Multifunctional Nanomaterials

**DOI:** 10.1021/acs.accounts.4c00206

**Published:** 2024-07-04

**Authors:** Adrian Moreno, Mika H. Sipponen

**Affiliations:** † Laboratory of Sustainable Polymers, Department of Analytical Chemistry and Organic Chemistry, Rovira i Virgili University, Tarragona 43007, Spain; ‡ Department of Materials and Environmental Chemistry, Stockholm University, SE-10691 Stockholm, Sweden; § Wallenberg Wood Science Center, Department of Materials and Environmental Chemistry, Stockholm University, SE-10691 Stockholm, Sweden

## Abstract

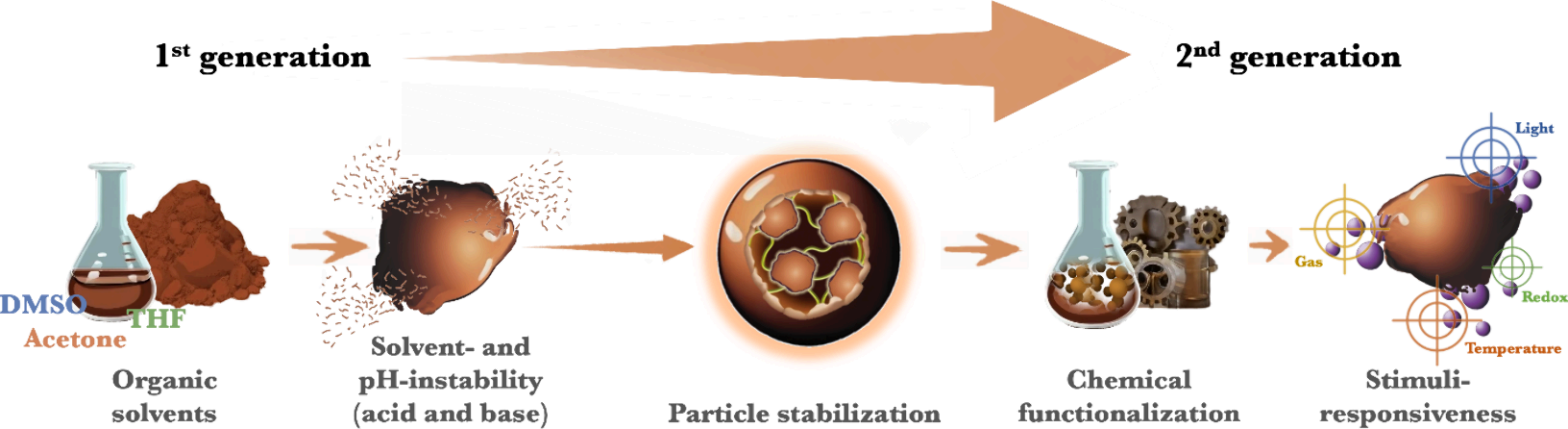

The increasing demand for polymeric materials derived from petroleum resources, along with rising concerns about climate change and global plastic pollution, has driven the development of biobased polymeric materials. Lignin, which is the second most abundant biomacromolecule after cellulose, represents a promising renewable raw material source for the preparation of advanced materials. The lucrative properties of lignin include its high carbon content (>60 atom %), high thermal stability, biodegradability, antioxidant activity, absorbance of ultraviolet radiation, and slower biodegradability compared to other wood components. Moreover, the advent of lignin nanoparticles (LNPs) over the last ten years has circumvented many well-known shortcomings of technical lignins, such as heterogeneity and poor compatibility with polymers, thereby unlocking the great potential of lignin for the development of advanced functional materials.

LNPs stand out owing to their well-defined spherical shape and excellent colloidal stability, which is due to the electrostatic repulsion forces of carboxylic acid and phenolic hydroxyl groups enriched on their surface. These forces prevent their aggregation in aqueous dispersions (pH 3–9) and provide a high surface area to mass ratio that has been exploited to adsorb positively charged compounds such as enzymes or polymers. Consequently, it is not surprising that LNPs have become a prominent player in applied research in areas such as biocatalysis and polymeric composites, among others. However, like all ventures of life, LNPs also face certain challenges that limit their potential end-uses. Solvent instability remains the most challenging aspect due to the tendency of these particles to dissolve or aggregate in organic solvents and basic or acidic pH, thus limiting the window for their chemical functionalization and applications. In addition, the need for organic solvent during their preparation, the poor miscibility with hydrophobic polymeric matrices, and the nascent phase regarding their use in smart materials have been identified as important challenges that need to be addressed.

In this Account, we recapitulate our efforts over the past years to overcome the main limitations mentioned above. We begin with a brief introduction to the fundamentals of LNPs and a detailed discussion of their associated challenges. We then highlight our work on: (i) Preparation of lignin-based nanocomposites with improved properties through a controlled dispersion of LNPs within a hydrophobic polymeric matrix, (ii) Stabilization of LNPs via covalent (intraparticle cross-linking) and noncovalent (hydration barrier) approaches, (iii) The development of an organic-solvent-free method for the production of LNPs, and (iv) The development of LNPs toward smart materials with high lignin content. Finally, we also offer our perspectives on this rapidly growing field.

## Key References

- Moreno, A.; Morsali, M.; Liu, J.; Sipponen, M. H. Access to Tough and Transparent Nanocomposites via Pickering Emulsion Polymerization using Biocatalytic Hybrid Lignin Nanoparticles as Functional Surfactants. *Green Chem.***2021**, 23, 3001–3014.^1^*This work describes the preparation of polystyrene and poly(butyl methacrylate) nanocomposites containing lignin nanoparticles with enhanced mechanical and UV-blocking properties by simple melting of polymeric latex dispersions obtained from radical polymerization of oil-in-water Pickering emulsions stabilized by hybrid lignin nanoparticles*.
- Moreno, A.; Liu, J.; Gueret, R.; Hadi, S. E.; Bergstrom, L.; Slabon, A.; Sipponen, M. H. Unravelling the Hydration Barrier of Lignin Oleate Nanoparticles for Acid- and Base-Catalyzed Functionalization in Dispersion State. *Angew. Chem. Int. Ed.***2021**, 60, 20897.^2^*This study shows that controlling the degree of esterification significantly improves the stability of hybrid lignin oleate nanoparticles in acidic and basic aqueous dispersions owing to the accumulation of acyl chains close to the particle surface producing a hydration barrier*.
- Pylypchuk, I.; Sipponen, M. H. Organic solvent-free production of colloidally stable spherical lignin nanoparticles at high mass concentrations. *Green Chem.***2022**, 24, 8705–8715.^3^*This work describes an organic solvent-free method for the production of lignin nanoparticles of poorly water-soluble lignins in the presence of sodium lignosulfonate. The lignin nanoparticle dispersions exhibit shear-thinning behavior and undergo gelation within well-defined pH and concentration regions*.
- Moreno, A.; Delgado-Lijarcio, J.; Ronda, J. C.; Cádiz, V.; Galià, M.; Sipponen, M. H.; Lligadas, G. Breathable Lignin Nanoparticles as Reversible Gas Swellable Nanoreactors. *Small*, **2023**, 19, 2205672.^4^*This study shows the preparation of gas-responsive lignin nanoparticles exceeding 75 wt % in lignin content. The reversible swelling behavior upon O*_*2*_*/N*_*2*_*bubbling of the particles was demonstrated for the fabrication of gas tunable nanoreactors for the synthesis of gold nanoparticles*.

## 1 Introduction

**Figure 1:**
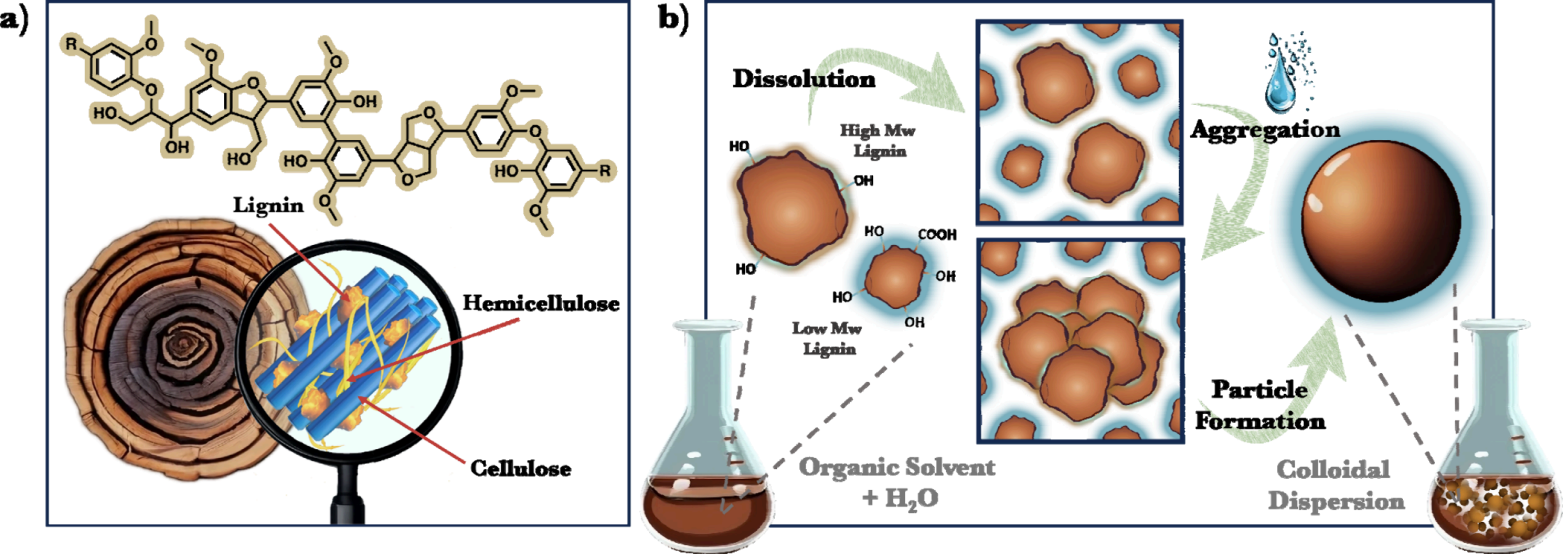
(a) Lignin distribution in lignocellulosic biomass and example of the lignin structure. (b) Schematic representation of colloidal self-assembly process of lignin into LNPs. Note: not drawn to scale.

Lignin is one of the promising renewable raw materials for the preparation of advanced materials.^5−8^ In nature, lignin reinforces plant cells by embedding cellulose and hemicellulose, adding rigidity to the cell walls and protecting against biological stresses.^9^ From a chemical point of view, once isolated from wood, lignin consists of amorphous, three-dimensionally branched aromatic molecules containing methoxy groups, aliphatic and phenolic hydroxyl groups, and some terminal carboxylic acid groups located at the side chains (Figure 1a). The structural differences between lignins depend on their botanical source and the extraction process from lignocellulosic biomass.^5,10,11^

The widespread interest in lignin-based advanced materials in various fields can be attributed to the successful development of colloidal lignin chemistry over the past decade, with lignin nanoparticles (LNPs) under the spotlight.^12−15^ LNPs are typically prepared via solvent-exchange methodology, where lignin is dissolved in an organic solvent, and poured rapidly or gradually into a water solution or vice versa.^12,13^ The formation of LNPs proceeds via aggregation of lignin, induced by hydrophobic interactions and π–π stacking of aromatic rings when the volume fraction of organic solvent is reduced. Other noncovalent interactions such as intra- and intermolecular hydrogen bonding and van der Waals forces contribute to the stabilization of the formed aggregates. Therefore, the formation of LNPs is essentially governed by the molecular size and (in)solubility of the lignin molecules in such a way that the stable particles have relatively more hydrophobic cores composed of higher molecular weight lignin molecules and surfaces consisting of relatively smaller lignin molecules enriched with hydrophilic groups (Figure 1b). This LNP formation via nucleation–growth mechanism has been validated by GPC and SEM analyses,^16^ while ^1^H liquid-state nuclear magnetic resonance spectroscopy has proved the presence of hydrophilic hydroxyl (aliphatic and phenolic), carboxylic acid, and methoxy groups at the surfaces of the LNPs arising mainly from the S- and G-units and β-O-4′ substructures.^17^ Here, it is important to note that the presence of carboxylic acid groups in their ionized form results in an increase in the surface charge of LNPs, which is crucial for their stabilization via electrostatic repulsion. Additionally, there are some cases where hemicelluloses can stabilize lignin particles.^11,18^ This stabilization of LNPs by attached polysaccharide chains is due to increased osmotic pressure when the particles approach each other, as the concentration of polysaccharide segments locally increases, causing a repulsive force. Recently, DFT calculations also support that the molecular structure of lignin strongly influences the formation of LNPs, so that flexible interunit linkages, specifically the β-O-4′ substructures, yield molecular folding resulting in intramolecular π–π stacking which presumably supports the assembly process.^19^

Solvents such as tetrahydrofuran, acetone, dimethyl sulfoxide, and ethanol are commonly used to dissolve lignin.^20,21^ However, they usually need to be combined with low amounts of water (3:1 w/w ratio) in order to achieve complete solubility of lignin before particle formation. Alternatively, it is possible to harness the partial solubility of lignin in polar organic solvents to prepare LNPs from specific lignin fractions. For instance, solvent fractionation of SKL with polar solvents such as ethanol offers the possibility to separate insoluble high molecular weight (MW) and soluble low MW lignin fractions, with the latter producing smaller LNPs. The high MW lignin fraction promotes a faster and more efficient dense packing via hydrophobic π–π stacking interactions. In the same manner, differences in the distribution of functional groups present on the surface of LNPs can also be detected since, for example, soluble and low molecular weight lignin fractions are usually more enriched with carboxylic acid groups. Although the solvent-exchange methodology is the most popular approach for the preparation of LNPs, aerosol technology is another alternative approach to prepare LNPs, in which solvent is vaporized, forming supersaturated lignin aerosol droplets that collapse into a spherical shape at the hydrophobic solvent–air interface.^22,23^ Other approaches, albeit less common, include the use of emulsion templates through self-driven encapsulation of hydrophobic compounds (oils)^24,25^ or precipitation of lignin by adjustment of pH which typically leads to the formation of irregular particles.^26^ For more information about the preparation of LNPs using either “dry” (aerosol technology) or “wet” (solvent exchange) processes, we direct the readers to the excellent and recent reviews.^12,13^

**Figure 2:**
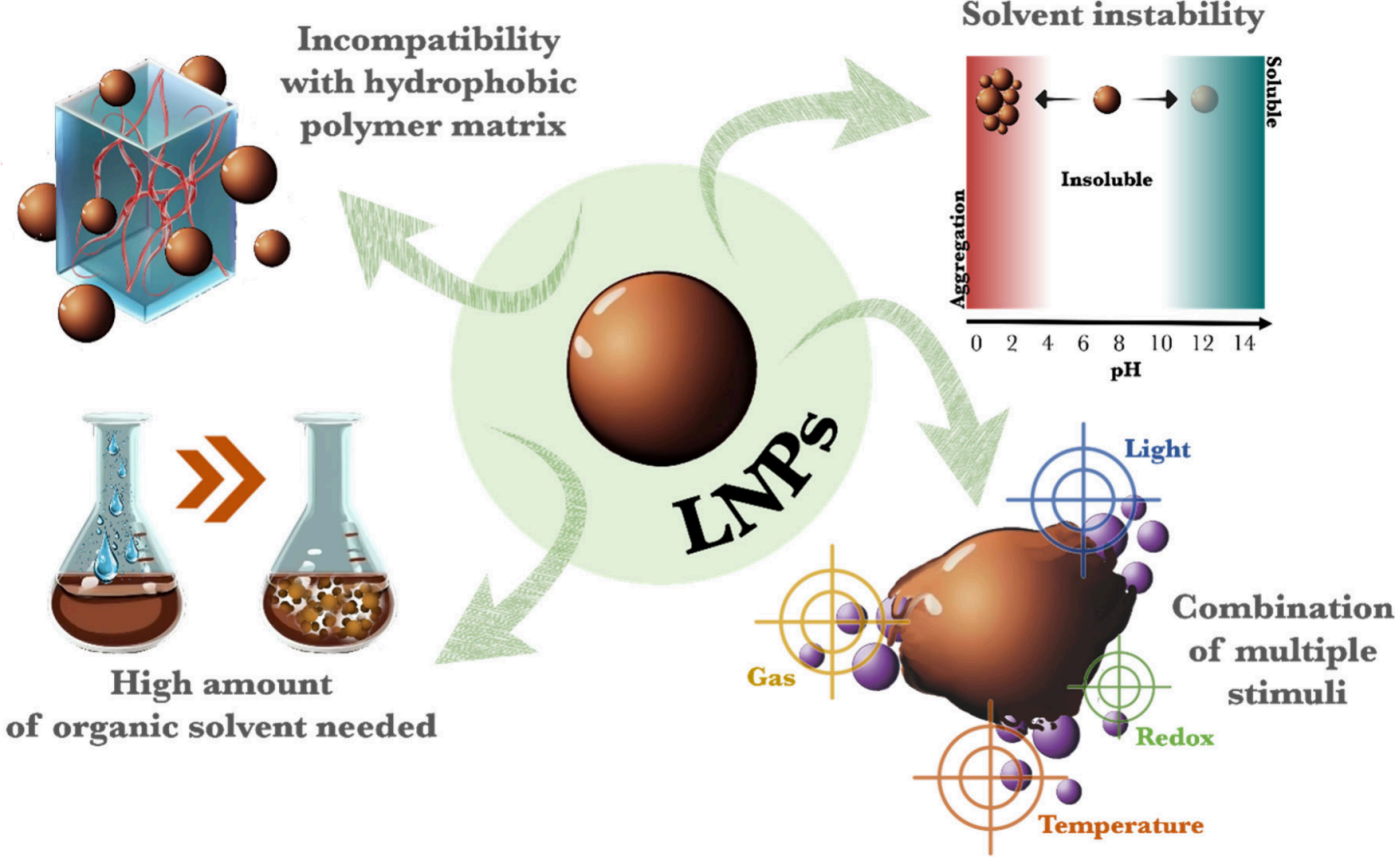
Current challenges associated with LNPs.

The attention that colloidal lignin materials have captivated is based on the superior properties of LNPs in contrast to bulk lignin. Among them, a well-defined spherical shape accompanied by the presence of negatively charged functional groups (phenolic hydroxyl and carboxylic acid), and a large surface area to mass ratio make them suitable for adsorption of positively charged compounds such as enzymes or polymers.^27,28^ In addition, LNPs resist aggregation in aqueous dispersions in neutral to slightly acidic pH owing to their submicrometer size and the electrostatic repulsion between the aforementioned negatively charged surfaces.^12,29^ In this regard, LNPs are able to circumvent challenges of crude lignins such as their poor interfacial binding within the polymeric matrix and aggregation during the preparation of lignin-based polymeric composites.^14,30^ However, LNPs also face some challenges such as (i) the use of a considerable amount of organic solvents for their production, which hinder their transfer from academia to industry, (ii) incompatibility with hydrophobic polymeric matrices when they are used as fillers for the preparation of polymeric composites, (iii) solvent instability, i.e., dissolution or aggregation in alkaline and acidic pH and organic solvents, and (iv) lack of complementary stimuli in the design of smart functional nanomaterials (Figure 2).

Over the last years, our group has focused on tackling the above-mentioned challenges in order to unlock and expand the potential of LNPs for different applications. In the next sections of this Account, we discuss our and other’s contributions in these frontiers. This Account is structured following the chronological developments carried out in our laboratory. We begin with a discussion on the different strategies to overcome the incompatibility of LNPs with hydrophobic polymeric matrices, followed by the current synthetic strategies for the stabilization of LNPs and their chemical functionalization in dispersion state. Thereafter, we introduce an alternative approach to prepare LNPs without the need for organic solvents and the preparation of stimuli-responsive LNPs with higher lignin content. Furthermore, we provide our perspectives on the upcoming challenges and opportunities in this rapidly growing field.

## 2 Dispersing LNPs into Hydrophobic Polymeric Matrixes

Synthetic polymeric nanoparticles (SPNPs) are widely used materials as reinforcing agents during the production of polymeric composites. Integration of these particles within the polymeric matrix results in synergic effects on different materials properties (e.g., electrical, mechanical, or catalytic, among others) that cannot be achieved from their constituent single components.^31−34^ Hence, given the aforementioned intriguing properties of LNPs, one of their most common applications is as fillers in the preparation of polymeric nanocomposites.^14^ In this way, LNPs have been combined with cellulose nanofibrils (CNF),^35,36^ poly(vinyl alcohol) (PVA),^37^ and chitosan,^38^ among others^39,40^ to produce polymeric nanocomposites with improved photothermal, UV shielding, mechanical, and antioxidant properties. Here, it is important to note that all the aforementioned cases have in common that the polymeric matrix is composed of a water-soluble or a hydrophilic polymer, allowing LNPs to be well dispersed and efficiently interact within the polymeric matrix. However, when the polymeric matrix comprises a hydrophobic polymer, the scenario drastically changes, as challenges related to inadequate interfacial binding within the polymeric matrix arise, resulting in LNP aggregation and phase separation processes.^41−43^ Consequently, the polymeric composites may not exhibit enhancement in properties, and in certain instances a decrease can be observed, notably in mechanical properties.

**Figure 3:**
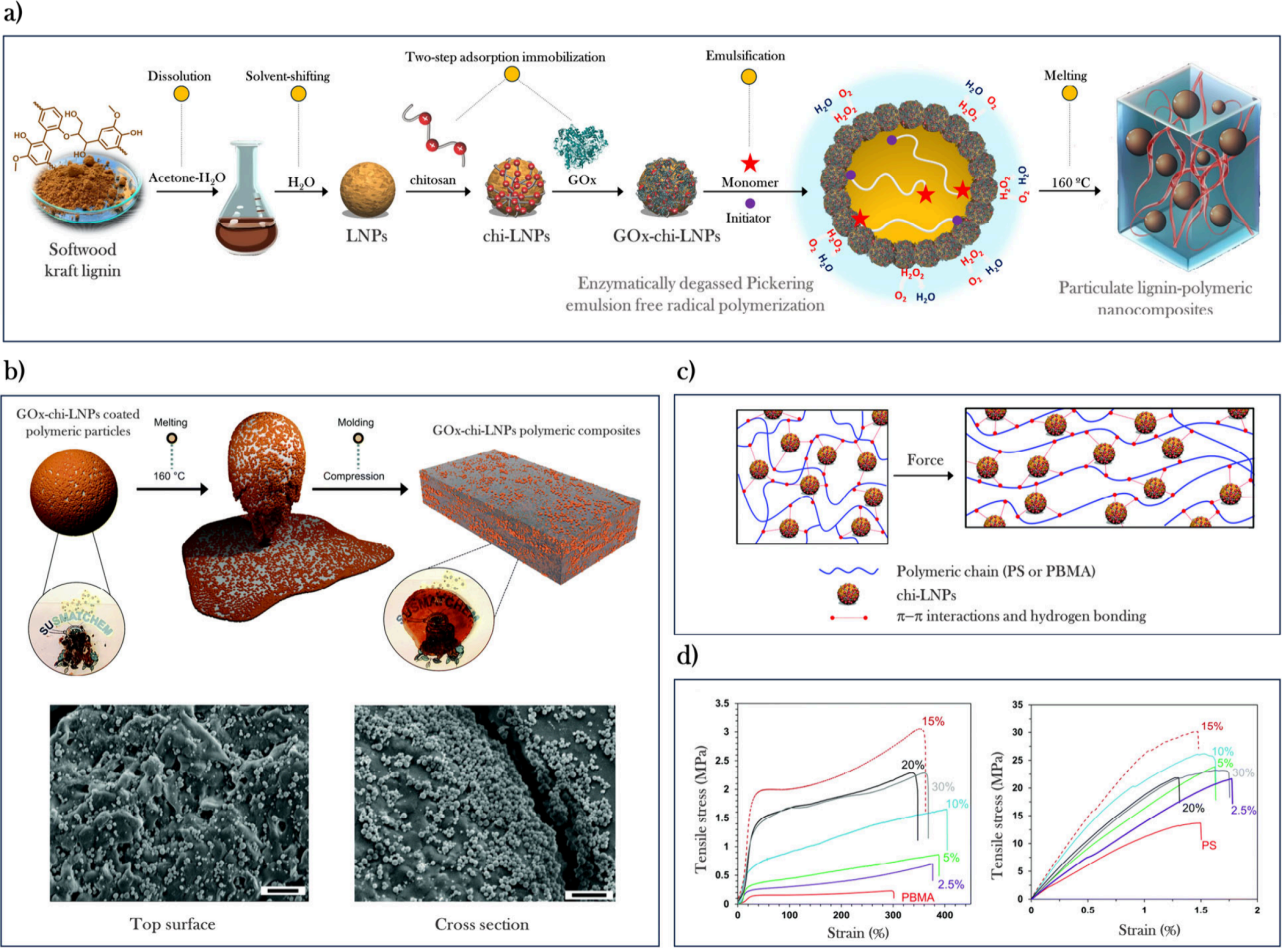
(a) Schematic illustration of the preparation of biocatalyst-loaded LNPs (GOx-chi-LNPs) and their application as functional surfactants in enzyme-degassed Pickering emulsion polymerization to produce particulate lignin-polymeric nanocomposites. (b) Schematic illustration of the preparation of the GOx-chi-LNPs-polymeric composites by the melting process and SEM micrographs of top and cross-sectional surfaces of PS-GOx-chi-LNP composite films. (scale bars: 1 μm). (c) Schematic illustration of the proposed interactions between hybrid LNPs with polymeric chains before and after deformation in tensile testing. Note: not drawn to scale. (d) Tensile stress–strain curves of PBMA and PS, and their composites with GOx-chi-LNP. Adapted from ref (1). Available under a CC-BY 3.0 DEED license. Copyright 2021 Royal Society of Chemistry.

In order to overcome this limitation, we reported a material-efficient method for the fabrication of hydrophobic polymeric composites that incorporated LNPs and improved mechanical, UV-shielding, and antioxidant properties.^1,44^ Our system is based on the fabrication of enzyme-coated LNPs and their application as functional surfactants for biocatalytically degassed radical polymerization of hydrophobic monomers in Pickering emulsions. After the polymerization, the latex dispersions were converted to hydrophobic polymeric composites with a homogeneous distribution of LNPs by a simple melting process (Figure 3a). The fabrication of the enzyme-coated LNPs involved a two-step adsorption process in which chitosan (chi) and glucose oxidase (GOx) were adsorbed onto LNPs to produce biocatalytic hybrid particles (GOx-chi-LNPs) capable of circumventing the oxygen inhibition of the radical polymerization process. The confirmation of the successful adsorption of chitosan and GOx into LNPs was evaluated based on dynamic light scattering measurements (DLS), with a gradual increment in particle size from 97 to 215 nm with associated reversal of the zeta potential from negative (−29 mV) to positive (+42 mV). These hybrid colloidal particles were used to stabilize hydrophobic monomer (styrene or butyl methacrylate)-in-water Pickering emulsions at a concentration of 9 g L^–1^, particles/monomers, while enabling efficient thermally initiated free radical or copper-catalyzed controlled radical polymerization in an open-air system showing the robustness of the system. After the polymerization, the analysis of the latex dispersions revealed polymeric beads efficiently covered by GOx-chi-LNPs. Melting of the dried polystyrene (PS) or poly(butyl methacrylate) (PBMA) latexes produced polymeric composite films with excellent distribution of the nonmelting lignin particles as fillers in the polymer matrix (Figure 3b). The evaluation of polymeric composites with different concentrations (wt %) of GOx-chi-LNPs for mechanical properties revealed a substantial improvement in toughness. Specifically, at 15 wt % of hybrid particles, toughness was boosted by a factor of 3.5 and 15 compared to pristine PS and PBMA, respectively (Figure 3d). We postulated that the enhancement in mechanical properties stems from both the effective dispersion of hybrid particles in the polymeric matrix and their favorable surface-area-to-mass ratio. Additionally, the effective noncovalent interactions within the matrix likely contribute by acting as sacrificial bonds, forming new bonds during deformation, thus explaining the positive reinforcing effect observed in our polymeric composites (Figure 3c). In addition to improving mechanical properties, the hybrid particles also conferred efficient UV-blocking and antioxidant properties to the polymeric composites, crucial for sectors like food packaging. However, potential safety issues arising from the migration process should also be considered, but so far we are limited to evidence from the antimicrobial activity of chi-LNPs.^45^ In summary, our approach not only integrated LNPs into hydrophobic polymeric systems but also enhanced mechanical properties while adding UV-blocking and antioxidant properties, overcoming a significant challenge in lignin-hydrophobic polymer composite preparation.

Continuing in the same direction, Kimiaei et al. also took advantage of the surfactant properties of LNPs to prepare cellulose-polycaprolactone (CNF-PCL) nanocomposites with improved mechanical properties.^46^ In their system, an aqueous CNF dispersion was combined with hydrophobic polycaprolactone (PCL) using LNPs as the emulsion stabilizer. The CNF-PCL films containing 10–30 wt % of LNPs exhibited a remarkable improvement in dry strength, showing around five to six times higher strain compared to the reference nanocomposites without LNPs. Additionally, the wet strength reached up to 87 MPa, significantly surpassing the previously reported wet strength of CNF cross-linked with tannic acid, epoxies, or multivalent metal ions, which ranged between 30 and 70 MPa. The superior properties of the nanocomposites were attributed to the capability of LNPs to form noncovalent bonds with both cellulose and PCL, thus serving as an interfacial compatibilizer. The ease with which this methodology can be applied to other hydrophobic polymers exemplifies the potential of LNPs in crafting hydrophobic polymeric nanocomposites with a favorable carbon footprint. More recently, Wang et al. also exploited the surfactant properties of LNPs in a seeded free-radical emulsion copolymerization of butyl acrylate and methyl methacrylate.^47^ In their approach, lignin was allylated prior to the formation of LNPs to include polymerizable allyl groups on the surface of LNPs. The resulting allylated-LNPs were then used as active interfacial-modulating surfaces to control emulsion polymerization, forming multienergy dissipative latex film structures with a lignin-dominated core (16% dry weight basis) via a simple casting method. The LNPs-integrated latex film demonstrated exceptional toughness exceeding 57.7 MJ m^–3^, achieved through an optimized allyl-terminated concentration of 1.04 mmol g^–1^. This enhancement in mechanical properties represents the most significant improvement reported in the literature so far. However, the necessity for solution-stage chemical modification of lignin could impede scalability and the transition to industrial processes.

## 3 Overcoming Solvent-Instability of LNPs: Access to Chemical Functionalization of LNPs in Dispersion State

Unlike modifying lignin in solution, focusing on chemical modification directly on solid particle surfaces could be more effective and open avenues to improve the compatibility of LNPs with polymeric matrices, as described in the previous section. However, enhancing the stability of LNPs under harsh conditions is necessary to develop advanced LNPs-based materials via acid/base catalysis and reactions in organic solvents. In this sense, chemical functionalization of LNPs in dispersion state has been viewed as a restricted area owing to the solubility of LNPs at pH > 10 due to ionization of the phenolic hydroxyl groups. There are also challenges in acidic conditions since LNPs have a point of zero charge and aggregate under acidic conditions at pH < 3 due to the protonation of their carboxylic acid groups.^48^ In addition, switching from aqueous to organic solvents either solubilizes the LNPs or leads to their aggregation. In this regard, the vast majority of functionalized LNPs necessitate the chemical modification of lignin before the particles are formed.

Pioneering works to overcome these considerable challenges include the work by Nypelö et al., who combined Kraft lignin with epichlorohydrin in a water-in-oil microemulsion to create intraparticle-cross-linked LNPs.^49^ The resulting LNPs exhibited strong resistance to dissolution when exposed to a highly alkaline environment at pH 13. Afterward, Mattinen et al. reported the use of laccases to achieve the stabilization of LNPs by means of a radical-mediated oxidative process, resulting in LNPs that are resistant to dissolution in organic solvents such as THF.^50^

Despite progress in particle stabilization, the chemical functionalization of LNPs in dispersion state has remained relatively less explored. The aforementioned methods relied on emulsion templates or enzyme-catalyzed cross-linking processes, effective only at low LNPs concentrations. The first chemical functionalization of LNPs in dispersion state was reported by Zou et al., who demonstrated a simple route to prepare internally cross-linkable epoxy-lignin hybrid particles.^51^ In their approach, an epoxy-cross-linker (bisphenol A diglycidyl ether, BADGE) was dissolved with lignin in an acetone:water (3:1 w/w) solvent mixture and hybrid particles with 10–40 wt % of cross-linker were formed following the solvent-exchange methodology. Thermally induced ring-opening reactions were demonstrated for intra- and interparticle cross-linking. The authors demonstrated that by using BADGE concentrations ≤20 wt %, it is possible to control the cross-linking within the particles, thus preserving their colloidal stability. The covalently stabilized particles remained intact even after being rinsed with aqueous acetone at a similar composition as that employed in particle production. Furthermore, these particles could be covalently functionalized via a base-catalyzed ring-opening reaction employing a quaternized epoxide, resulting in particles with a surface net charge that responds to pH (positively charged at pH < 5; negatively charged at pH > 5) Additionally, the hybrid particles containing 30 wt % BADGE were utilized as thermally curable particulate adhesives, exhibiting dry strength comparable to and wet strength surpassing that of a commercial epoxy adhesive. Overall, these findings suggest that adding a cross-linker during LNPs’ supramolecular assembly is a successful strategy for achieving stable and functionalized LNPs, a method later extended by our group and others.^52,53^

**Figure 4:**
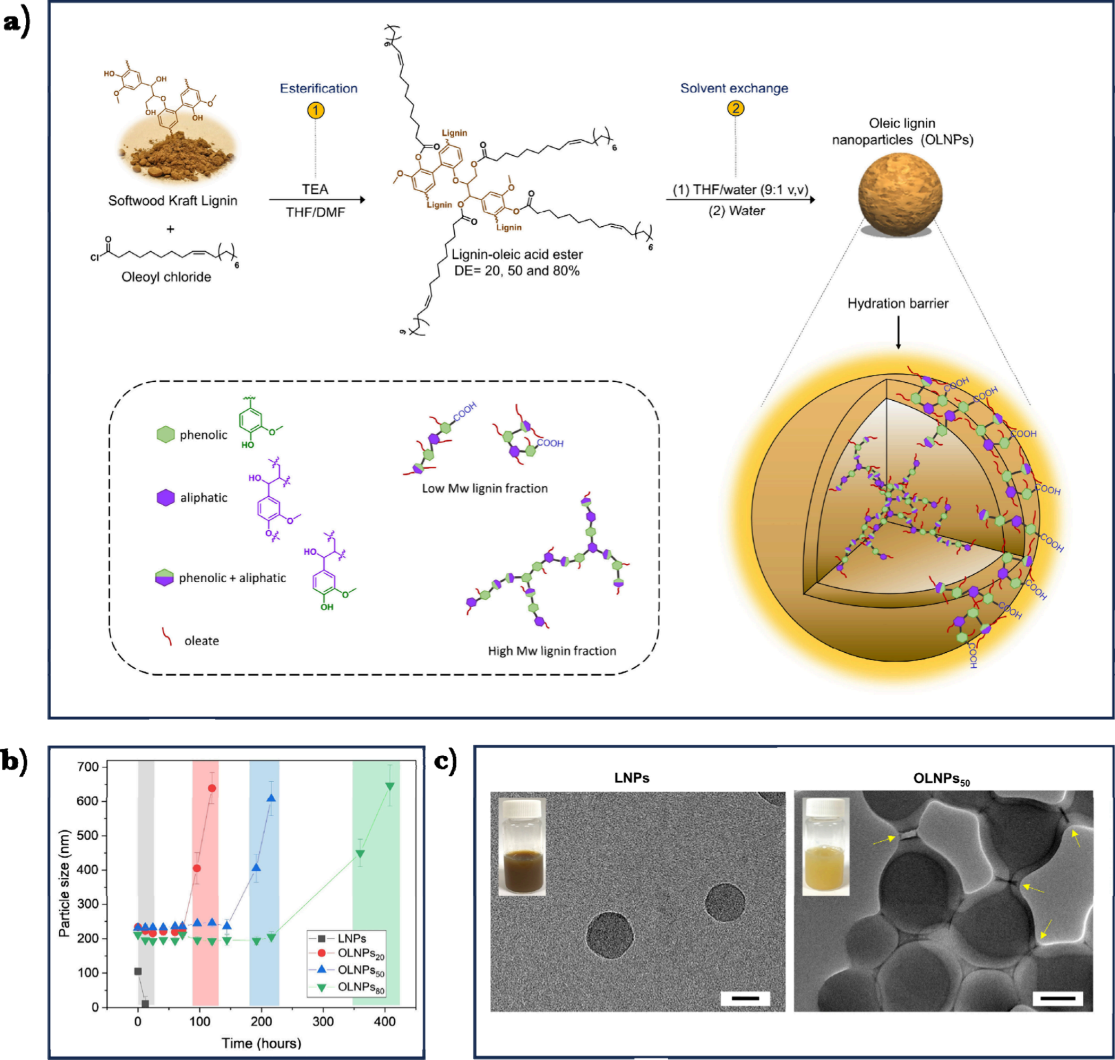
(a) Illustration of the preparation of oleic lignin nanoparticles (OLNPs): 1) Base-catalyzed esterification of SKL with oleoyl chloride; 2) production of OLNPs via solvent exchange precipitation from lignin-oleic acid esters. The orange color around the OLNP surface indicates the hydration barrier produced by the oleate chains. (b) Evolution of particle size for LNPs and OLNPs at pH 12.0. The colored dashed sections indicate the time-dependent aggregation/dissolution of different particles. (c) TEM images of LNPs and OLNPs_50_ (scale bar: 100 nm). Inset digital images correspond to LNPs and OLNPs colloidal dispersions. Adapted from ref (2). Available under a CC-BY 4.0 DEED license. Copyright 2021 Wiley.

Inspired by the preceding work, our team began development of environmentally friendly alternatives to BADGE. Our approach involved esterifying lignin with an oleoyl fatty acid derivative to achieve lignin-oleate, which could then be cross-linked using free radical chemistry.^2^ In this way, oleic lignin nanoparticles (OLNPs) were prepared via solvent-exchange methodology from lignin oleates with different degrees of esterification (DE = 20%, 50%, and 80%) (Figure 4a). Initially, we speculated that oleic fatty acid chains would be restricted to the inner core–shell—core of the particle—of OLNPs, and internally stabilized particles would be feasible to obtain via the radical cross-linking of the double bond present in the unsaturated oleic chain. DLS analysis of OLNPs revealed no significant differences in particle sizes among the three OLNPs (around 200 nm). However, direct comparison between LNPs and OLNPs pointed out a significant difference in particle sizes (100 nm vs 200 nm, respectively), which was attributed to the effect of unsaturated oleic chains that would hamper an efficient molecular dense packing during the self-aggregation process. Stability studies under basic and acidic conditions (pH = 12 and pH = 2) revealed unprecedented stability for OLNPs without thermal curing, which increased with the DE of the lignin-oleic precursor. OLNPs_20_ (DE = 20%) remained colloidally stable for 48 h under basic conditions while OLNPs_80_ (DE = 80%) exhibited stability for more than 100 h (Figure 4b). Based on these observations and supported by TEM imaging of core–shell structures of OLNPs (Figure 4c), we hypothesized that the exceptional stability of the OLNPs stemmed from a hydration barrier created by the oleic fatty acid chains collapsed on the particle surface.

**Figure 5:**
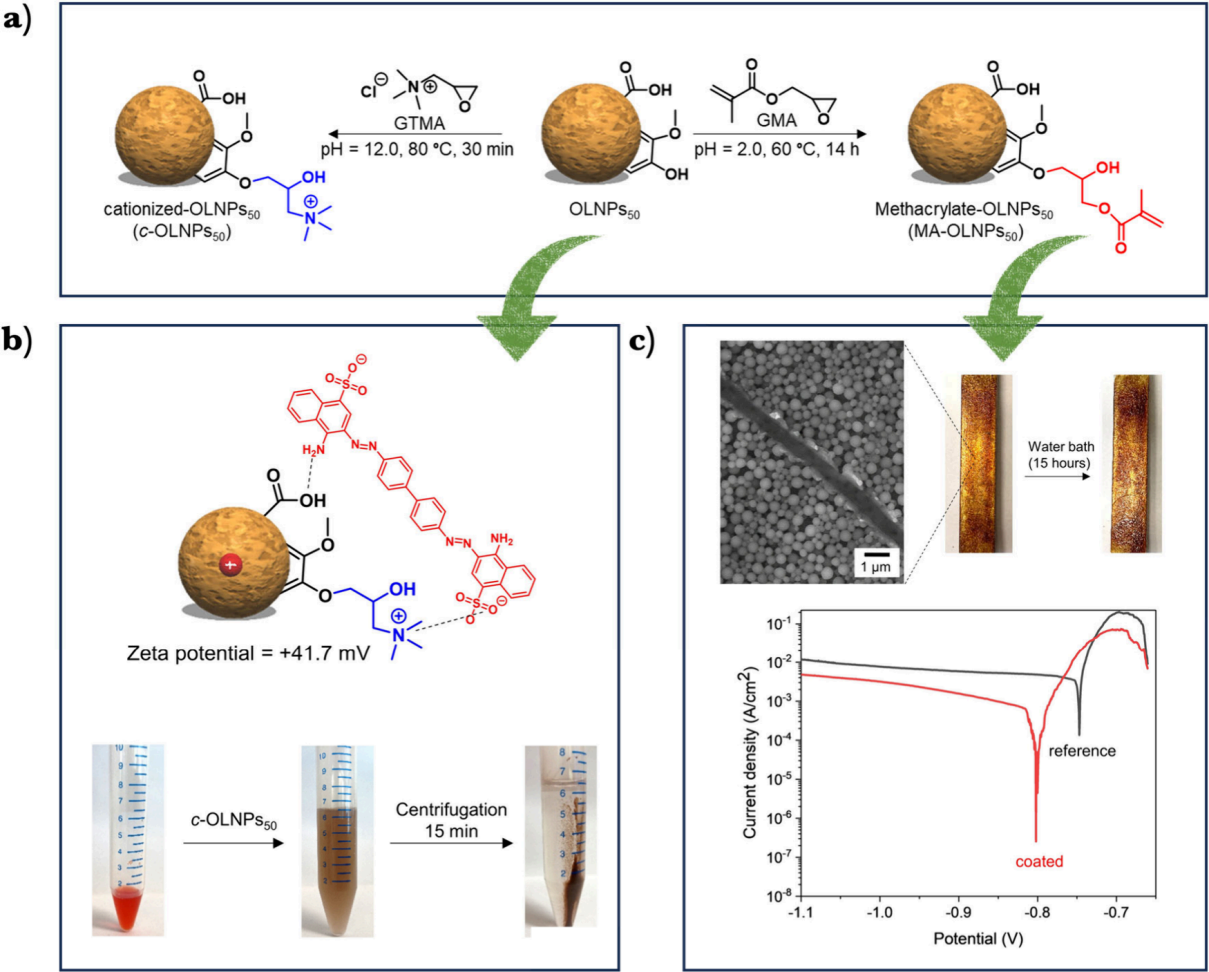
(a) Surface covalent functionalization of OLNPs_50_: (a) left: base-catalyzed ring-opening of GTMA under basic conditions (pH 12.0), right: acid-catalyzed ring-opening reaction of GMA under acidic conditions (pH 2.0). OLNPs_50_ was used as a nucleophile for oxirane ring-opening. (b) Application of *c*-OLNPs_50_ in dye adsorption in aqueous solutions: Illustration of the electrostatic interaction between *c*-OLNPs_50_ with negatively charged Congo Red, and digital images of dye removal from aqueous solutions. (c) Application of MA-OLNPs_50_ as an anticorrosion coating for metal surfaces: SEM image of a diagonally scratched surface of MA-OLNPs_50_-coated Al specimen, and digital images before and after the exposure of cured MA-OLNPs_50_-coated Al specimen to saline water (5% NaCl) for 15 h. Potentiodynamic polarization curves (Tafel plots) of coated (MA-OLNPs_50_-coated Al, red line) and noncoated aluminum substrates (reference, black line) after exposure to a 5% NaCl solution at 25 °C for 15 h. Adapted from ref (2). Available under a CC-BY 4.0 DEED license. Copyright 2021 Wiley.

As previously mentioned, the formation of LNPs is influenced by molecular size distribution and hydrophobicity of lignin. Esterification of lignin with long fatty acids like oleic acid (C_18_) enhances its hydrophobicity and alters its structure. In this context, we proposed that high molecular weight lignin oleate molecules reside in the particle interiors, while low molecular weight esters containing hydrophilic carboxylic acid groups are oriented toward the hydrophilic surfaces, exposing them to the water phase. This arrangement prompts the hydrophobic effect, causing the oleate chains in low molecular weight fragments to collapse and associate at the surface, minimizing exposure to water (Figure 4a). Consequently, OLNPs display charged surfaces, where the deposited oleate chains form an effective hydration barrier that retards the ionization of phenolic groups under alkaline conditions and protonation of carboxylic groups in acidic media. Encouraged by these findings, we also conducted, for the first time, covalent functionalization of non-cross-linked LNPs in the dispersion state via base- and acid-catalyzed ring opening reactions (Figure 5a). Methacrylated OLNPs (MA-OLNPs) were utilized to create anticorrosive coatings for aluminum. The curing of MA-OLNPs resulted in a particulate coating that significantly reduced the corrosion current density (CCD) by 3 orders of magnitude, providing effective corrosion protection (Figure 5c). Additionally, the cationized OLNPs (*c*-OLNPs) were demonstrated as fast and effective pH-switchable adsorbents for water treatment (Figure 5b). More recently, our group reported an alternative methodology that involves the preparation of hydroxymethylated lignin nanoparticles (HLNPs) followed by a catalyst-free hydrothermal curing to trigger internal cross-linking reactions.^54^ In addition to allowing for dispersion state modification of the HLNPs, this methodology preserves the phenolic groups that are key functionalities defining biodegradability, redox activity, and antimicrobial properties of lignin. In a follow-up study, HLNPs were used to adsorb phospholipase D, allowing repeated use of this expensive enzyme over four cycles of transformation of phospholipids to polar headgroup-modified derivatives.^55^ This approach simplifies the use of LNPs in enzyme immobilization; previously enzyme-coated LNPs have been stabilized by encapsulation in calcium alginate beads for instance,^39^ or cationic LNPs coated with chitosan.^44^

Both strategies outlined above, using cross-linkers during self-assembly or providing a hydration barrier from fatty acids, are crucial for obtaining functionalizable LNPs and enabling access to lignin-based advanced materials. Intrigued by combining the synergies of these strategies, our group recently reported the use of Urushi (oriental lacquer) as a sustainable component to achieve stabilization of LNPs via an internal cross-linking process and hydration barrier.^56^ Hybrid particles containing ≤25 wt % Urushi exhibited stability following the thermally triggered cross-linking of its unsaturated hydrocarbon chains, attributed to Urushi’s deposition in the inner core of the particles. Conversely, hybrid particles with Urushi content >25 wt % showed enhanced stabilization via thermal interparticle cross-linking process effect owing to the surface exposure of Urushi’s hydrophobic chains. These particles demonstrate a great potential to prepare particulate coatings to protect wood from water under harsh conditions such as extreme pH.

## 4 Organic-Solvent-Free Wet Process: A Paradigm Shift in the Preparation of LNPs

**Figure 6:**
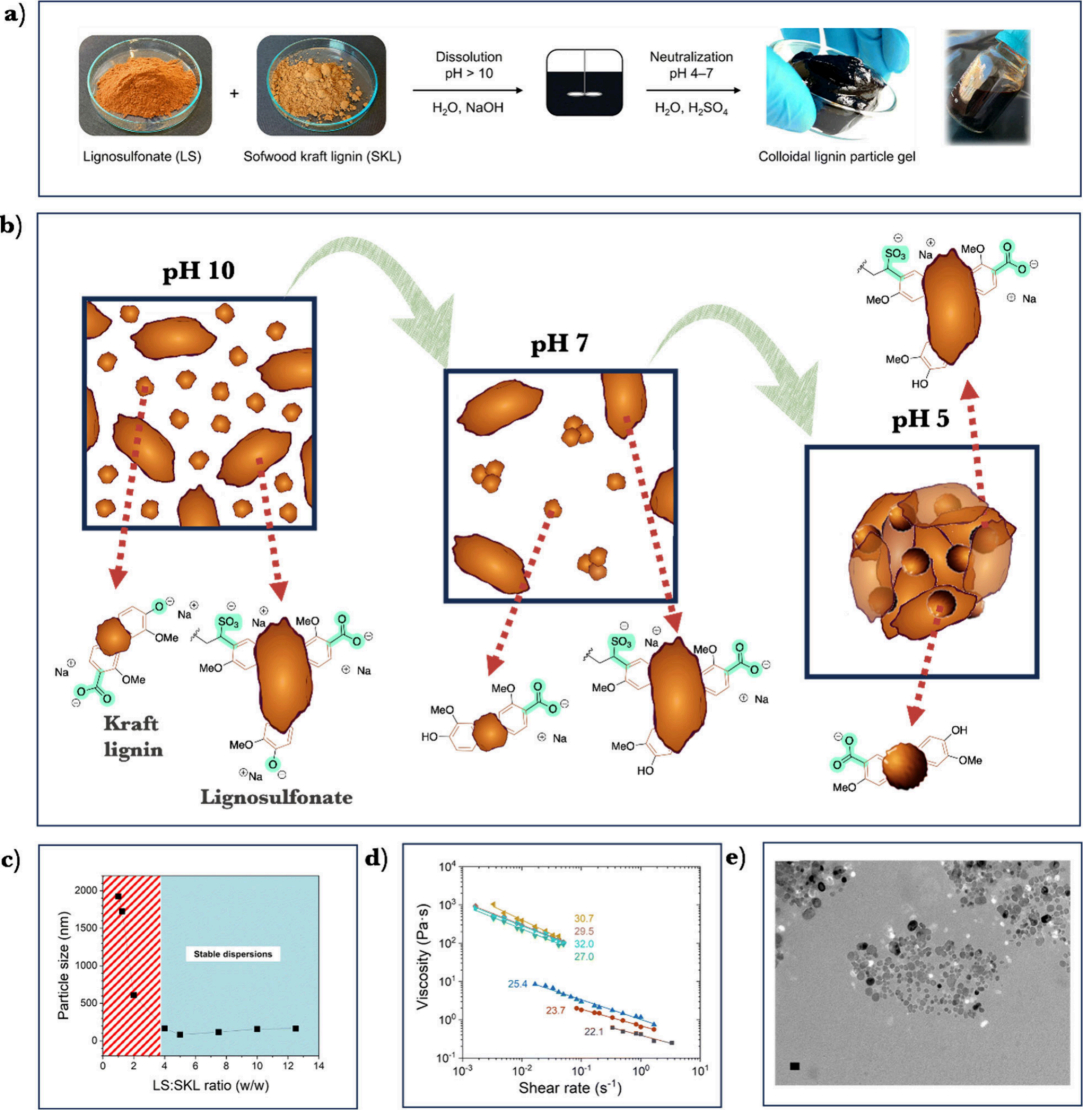
(a) Preparation of micellar particle gels and colloidal dispersions from sodium lignosulfonate (LS) and poorly water-soluble lignin such as softwood kraft lignin (SKL, pictured). (b) Schematic model of formation of micellar particles of lignosulfonate in the presence of softwood kraft lignin or other lignin grades poorly soluble below neutral pH. (c) Effect of LS:SKL mass ratio on particle size (hydrodynamic diameter, Z-average values based on DLS) and observed colloidal stability of the dispersions. (d) Rheological properties of colloidal lignin gels. Dependency of dynamic viscosity of LS-SKL (5:1 w/w) dispersion on total lignin concentration, expressed as wt %, while maintaining a constant pH 4.8. (e) Transmission electron microscopy (TEM) image of LS + SKL colloidal dispersion (5:1 w/w) (scale bar: 100 nm). Adapted from ref (3). Available under a CC-BY 3.0 DEED license. Copyright 2023 Royal Society of Chemistry.

Formation of LNPs by adding a nonsolvent (water) into a lignin solution in organic solvent or vice versa causes the formation of spherical particles via hydrophobic-induced aggregation of lignin (Figure 1b).^12^ Despite ongoing efforts, when it comes to the scale-up of these production processes, critical challenges remain to be overcome. For instance, solvent-exchange methodology lacks cost-efficient methods for the organic solvent recovery as the techno-economic assessments have shown.^21^ In addition, the range of concentrations at which LNPs can be produced as colloidally stable dispersions is limited to ∼2 wt %. Meanwhile, the aerosol technologies require a careful evaluation of the risks involved in the production, transportation, and handling of dry LNP powders.^22^ The contributions described in sections 2 and 3 were achieved with LNPs and hybrid LNPs prepared via the solvent-exchange methodology, which still face the aforementioned challenges. To address this challenge, our next step was to develop a robust method for the production of LNPs without the need of organic solvent. In this context, we proposed an approach that relies on the combination of two lignins with different aqueous solubility, as is the case with the two most important technical lignins: the poorly water-soluble softwood kraft lignin (SKL) and water-soluble sodium lignosulfonate (SL).^3^ Our methodology involves the dissolution of both LS and SKL in aqueous alkali (pH > 10), and the adjustment of the pH to slightly acidic (pH = 5.5) gives rise to a free-flowing micellar solution or gel as shown in Figure 6a. From a mechanistic point of view, as the pH decreases, poorly water-soluble lignins like SKL gradually precipitate and form spherical nuclei through hydrophobic interactions, while also associating with LS. Sulfonate groups of LS prevent molecular close packing and maintain a loosely packed micellar structure due to repulsive electrostatic interactions (Figure 6b). Systematic studies demonstrated that a 4:1 mass ratio of LS:SKL is the minimum requirement to obtain stable colloidal dispersions, while a 5:1 mass ratio produces the lowest particle size of 82 nm (Figure 6c). Instead of SKL, it is possible to use other poorly water-soluble lignins such as organosolv lignin (OLS) or soda lignin (SL). Unlike aqueous–organic solvent-based methods discussed previously, where lignin concentration is limited to 2 wt % to prevent particle size increase and subsequent agglomeration,^16^ the hydrodynamic diameter of the colloidal particles did not increase but decreased as the concentration of lignin increase from 2 wt % to 14 wt % at a fixed ratio of LS:SKL. This fact can be attributed to increased viscosity of the system and the resulting shear forces that effectively counteract the particle growth. This method notably extends the working window for lignin particle concentrations compared to prior methods.^12^

Rheological experiments revealed a distinct gelation point dependent on lignin concentration and pH (Figure 6d). Specifically, lignin concentrations around 26 wt % at pH 5–6 promote the formation of a continuous particulate network based on intra- and intermolecular interactions of the two lignin types. TEM images of the colloidal dispersions revealed spherical particles (25 nm) sensitive to the beam exposure, proving the micellar nature of the particles internally stabilized by hydrophobic interactions (e.g., π–π stacking) and externally by repulsive electrostatic interactions arising from the sulfonate groups (Figure 3e). Overall, in comparison to traditional methods, the key advantages of this approach include the elimination of organic solvents, the ability to operate at high concentrations (up to ∼50 wt %), and the simplicity of preparing shear-thinning lignin nanoparticle gels with self-supporting properties. Conversely, the softness of the micellar particles distinguishes them from the denser LNPs, indicating potential divergence in paths toward various different applications.

## 5 Unlocking the Potential: Toward Stimuli-Responsive, Photonic, and Circular LNPs

**Figure 7:**
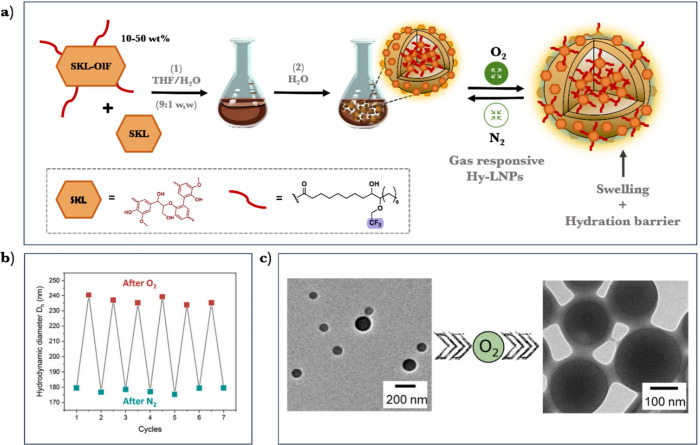
(a) Schematic illustration of the preparation of SKL-OlF-SKL hy-NPs and their gas-responsive behavior: 1) codissolution of SKL-OlF and SKL in tetrahydrofuran/water (9:1, w/w). 2) Gradual coprecipitation of SKL-OlF and SKL against water to form SKL-OlF-SKL NPs (hy-LNPs). (b) reversible changes in the hydrodynamic diameter (Dh) of hy-LNPs_30_ upon repeated cycles of alternating O_2_/N_2_ bubbling. (c) Transmission electron microscopy (TEM) images of hy-LNPs_30_ before and after the exposure to O_2_. Adapted from ref (4). Available under a CC-BY 4.0 DEED license. Copyright 2023 Wiley.

Stimuli-responsive materials, sometimes referred to as “smart” materials, have the ability to “sense” external stimuli such as pH, light, gas, or temperature and translate it into an observable response based on physiochemical changes.^7,8^ Regarding stimuli-responsive nanomaterials, such as polymeric nanoparticles, most efforts have focused on imparting a “programmable” degradation by introducing labile chemical groups (e.g., acetal, disulfide, etc.) to develop advanced drug delivery platforms.^57,58^ Stimuli-responsive LNPs have been explored toward drug delivery systems that harness the inherent properties of lignin. Dai et al. combined UV-blocking properties of lignin and the temperature responsiveness of poly(*N*-isopropylacrylamide) to develop temperature-responsive LNPs able to deliver on-demand *trans*-resveratrol, a light-sensitive drug.^59^ Another impressive effort is the work reported by Qian et al. where the well-known surfactant properties of lignin were combined with the ability of poly(dimethylaminoethyl acrylate) to interact with CO_2_ and N_2_ to develop reversible emulsifiers for Pickering emulsions processes.^60^ While these works exemplify the synergistic integration of lignin with stimuli-responsive functionalities, they typically require polymer grafting, resulting in a low lignin content (25 wt %) in the final material. In this sense, our group contributed with an alternative methodology to prepare gas (O_2_/N_2_)-responsive LNPs exceeding 75 wt % of lignin mass content.^4^ Our approach involves the solvent-exchange of SKL in the presence of a fluorinated lignin oleic acid ester (SKL-OlF) resulting in the formation of hybrid-LNPs (Figure 7a). The coaggregation of unmodified lignin with hydrophobic lignin derivatives resulted in the formation of hybrid particles, where the inner core is composed of the association and collapse of the more hydrophobic fragments.

Consequently, hy-LNPs containing SKL-OlF content ranging from 10 to 50 wt % exhibited reproducible reversible swelling behavior upon exposure to O_2_/N_2_, with a volume increase of approximately 35% (Figure 7b). This change in volume also led to a morphological shift from spherical to core–shell (Figure 7c). The swelling behavior and change in the morphology were ascribed to the effective interaction of O_2_ with C–F, promoting a decrease in the hydrophobicity of the fluorine oleate chains. Polarity changes prompt lignin-fluorinated oleic chains to migrate from the inner part to the particle surface, increasing particle swelling and enhancing stability under acidic conditions (pH < 2.5). We also showcased the potential of these LNPs as tunable gas nanoreactors for preparing gold-lignin hybrid nanoparticles. This approach offers exciting prospects for designing advanced nanomaterials based on LNPs, potentially serving as catalytic vessels for asymmetric chemical reactions.

Building on the regulated assembly of lignin particles, photonic lignin materials exhibit unique optical properties, including structural coloration, due to their periodic arrangement of nanoscale components. First demonstrated by Wang and co-workers,^61^ photonic lignin materials are gaining traction due to their ability to produce individual and rainbow colors as an alternative to photonics prepared from synthetic polystyrene latex particles.^62,63^ These materials hold great potential for various applications from biomedicine to environmental monitoring.

## 6 Summary and Outlook

In this Account, we have summarized the different approaches to overcome the main challenges associated with LNPs and highlighted our contributions to the field. Despite the exciting progress achieved, challenges still exist. First, the preparation of hydrophobic nanocomposites remains restricted to the use of low amounts of LNPs (15–30 wt %), relying mainly on acrylic monomers. In this sense, stabilized LNPs would be crucial to explore routes such as reverse emulsion processes, where the stability of the particles in organic solvent would promote the interactions within the polymeric matrix, allowing for increasing the concentrations of LNPs. Second, there are currently two main methodologies for the stabilization of LNPs: (i) entrapment of a cross-linker during the self-assembly process and (ii) formation of hydration barrier provided by fatty acids. Both strategies have allowed the chemical modification of LNPs in aqueous dispersion state under acidic and basic conditions. However, so far there are no examples of chemical functionalization of LNPs in organic solvents. Therefore, future works should assess the possibility to conduct chemical reactions with LNPs in green organic solvents. Among them, polymerization-induced self-assembly (PISA) processes would be of broad interest to obtain hybrid nanostructures with multiple morphologies based on LNPs, possibly allowing the access to advanced materials such as nanomotors where morphology control is crucial. Third, harnessing the valuable properties of lignin along with added stimuli-responsive functionalities is worth investigating to develop advanced materials with a favorable carbon footprint. However, thus far, these systems are limited to the introduction of a single stimulus, constraining their range of application. In this sense, the next generation of stimuli-responsive LNPs should address the introduction of multiple stimuli in a predictable manner. If successful, these advancements will allow for a “programmable” control of various stimuli and even their combination, enabling the development of complex cascade processes that mimic biological systems. First steps have already been taken with the development of multistimuli-responsive lignin microcapsules for the delivery of pesticides,^64^ but much effort introducing complementary stimuli is still needed. Such advancements could be of interest for the creation of advanced drug delivery systems based on LNPs (e.g., nanotheranostics). It is important to note that since some of the presented materials can function at the interface between material science and biology, evaluating their biodegradability, toxicity, and recyclability still requires more understanding and effort. For example, recycling our lignin-polymeric composites (section 2) could be challenging, involving basic extraction to solubilize the biocomponents. Additionally, the degradation of fluorinated lignin esters (section 5) could release small fluorine synthons into the environment. The biodegradability of these systems also remains a challenge, as chemical modification of lignin is expected to affect its biodegradation, given that free phenolic hydroxyl groups are the primary sites of enzymatic degradation of lignins in nature.^65^ Therefore, it is clear that further research is necessary to elucidate the end-of-life and environmental impacts of lignin-based nanomaterials in various emerging applications. Last but not least, we hope that this Account will inspire researchers to develop new methodologies aimed at maximizing and unlocking the potential of LNPs across disciplines, including chemical biology and materials science.
